# *Mycobacterium ulcerans* Disease, Peru

**DOI:** 10.3201/eid1403.070904

**Published:** 2008-03

**Authors:** Humberto Guerra, Juan Carlos Palomino, Eduardo Falconí, Francisco Bravo, Ninoska Donaires, Eric Van Marck, Françoise Portaels

**Affiliations:** * Universidad Peruana Cayetano Heredia, Lima, Perú; † Institute of Tropical Medicine, Antwerp, Belgium; ‡ Instituto Nacional de Salud del Perú, Lima, Perú; § University of Antwerp, Antwerp, Belgium

**Keywords:** Mycobacterium ulcerans, Buruli ulcer, Peru, Amazon, research

## Abstract

Eight adult patients (ages 18–58, 5 women) with Buruli ulcer (BU) confirmed by at least 2 diagnostic methods were seen in a 10-year period. Attempts to culture *Mycobacterium ulcerans* failed. Five patients came from jungle areas, and 3 from the swampy northern coast of Peru. The patients had 1–5 lesions, most of which were on the lower extremities. One patient had 5 clustered gluteal lesions; another patient had 2 lesions on a finger. Three patients were lost to follow-up. All 5 remaining patients had moderate disease. Diverse treatments (antituberculous drugs, World Health Organization [WHO] recommended antimicrobial drug treatment for BU, and for 3 patients, excision surgery) were successful. Only 1 patient (patient 7) received the specific drug treatment recommended by WHO. BU is endemic in Peru, although apparently infrequent. Education of populations and training of health workers are first needed to evaluate and understand the full extent of BU in Peru.

## Abstract 2

Eight adult patients with Buruli ulcer were seen in a recent 10-year period.

Buruli ulcer (BU), a chronic ulcerative disease, has been observed in many tropical areas, but patients have usually come from Africa and Australia (*1*–*6*). Cases were also described in the Americas, mostly in French Guiana (*3*,*6*). A few cases from Surinam have also been recorded in French Guiana, and 8 cases have been reported in Mexico since 1953 (*3*,*6*).

In 1969, the first 2 cases from Peru were reported (*7*). A new case was reported in 1988, along with a redescription of the first 2 cases (*8*). From 1996 through 2005, 8 additional cases, which we describe here, were found in Peru (Figure 1).

**Figure 1:**
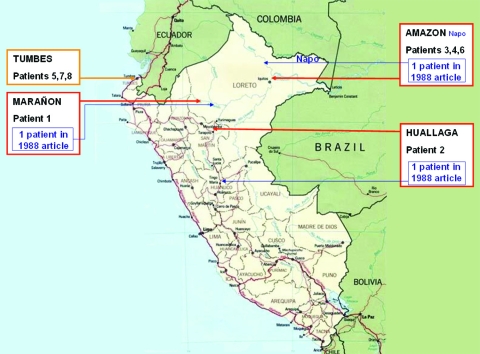
Map of Peru showing the locations where 8 Buruli ulcer patients were probably infected. Red, Peruvian River basin; gold, coastal area.

## Material and Methods

We conducted a descriptive, retrospective survey of patients seen by members of the Instituto de Medicina Tropical Alexander von Humboldt in Lima and Iquitos. We requested referrals of new patients from other areas. The total of 8 cases occurred from 1996 through 2005.

### Patients

We used a collective data sheet proposed by the World Health Organization (WHO) (*9*) to assess the magnitude and severity of the disease and to collect data from patients. We also obtained information from medical records. Patients who had a history of chronic ulcer and who had at least 2 different positive laboratory tests were included in this study (*10*). All the patients gave consent verbally. The publication was approved by the Human Protections Administration Office for Human Research Protection of the Universidad Peruana Cayetano Heredia (SIDISI code 52467).

### Smears and Tissue Collection

Smears obtained by scalpel or swab were prepared with material from the necrotic base and undermined edges of the lesions and were stained with Ziehl-Neelsen. Skin biopsy samples were excised and cut into >2 portions. At least 1 portion was fixed in 10% formalin and processed for histologic examination at the pathology laboratory of the Hospital Nacional Cayetano Heredia in Lima. The other portions, minced further, were inoculated after decontamination onto Löwenstein-Jensen medium in Lima as described previously (*10*); the rest was placed in a semisolid transport medium (*11*) and sent to the Institute of Tropical Medicine, Antwerp, for culture and PCR testing (*10*).

## Results

We found 8 cases of BU from 1996 to 2006. Case characteristics are indicated in Table 1. Five patients came from the Peruvian rainforest, the likely place of infection. Two patients reported close contact with water in the Marañón (patient 1) and Huallaga (patient 2) River basins. Three patients (patients 3, 4, and 6) lived close to Iquitos, a city on the Amazon River. One patient (patient 5) had briefly visited a swampy area in the north coast of Peru (Tumbes), and 2 other patients (patients 7 and 8) lived in the same area. All of the areas described are warm and humid. The age range at diagnosis was 18–58 years, with a male to female ratio of 3:5. No case patients had a medical or family history of tuberculosis or leprosy. The time between onset of illness and being seen by a physician was 1–8 months. Four patients noticed a nodular lesion before ulceration occurred. All patients had ulcers with typical undermined edges. The site of involvement in our patients was on the extremities, but 1 patient had gluteal lesions. The median number of lesions per patient was 2. Three patients (patients 1, 2, and 5) were lost to follow-up at an early stage, soon after diagnosis, and their disease course was unknown.

**Table 1:** Case descriptions of Buruli ulcer, Peru*

Patient no.	Geographic origin	Age, y/sex	Patient delay, mo	Localized pain	No. of lesions	Sites	Size of main lesion, cm	Treatment
1	Marañón	18/M	8	Positive	2	Left knee	7 × 6	Lost to follow-up, no treatment
2	Huallaga	22/F	1	Positive	1	Left thigh	2 × 3	Lost to follow-up, no treatment
3	Iquitos	54/M	2	Positive	2 (right knee earlier, larger)	Both knees	6 × 7	Antituberculous drugs and herbal medicines
4	Iquitos	58/F	8	Positive	5 (in a single group)	Left gluteal region	12 × 16 (sum of all 5)	Anti-*Leishmania* and herbal medicines
5	Tumbes	46/F	8	Positive	2 (1 lesion was a scar)	Left foot	5 × 6	Lost to follow-up, no treatment
6	Iquitos	21/F	3	Positive	1	Right thigh	5 × 5	Antituberculous drugs (regimen 1) and surgery
7	Tumbes	34/F	2	Positive	4	Right thigh and leg	6 × 8	WHO BU antibiotics and surgery
8	Tumbes	45/M	1	Positive	2	Right middle finger	8 × 2	Antituberculous drugs (regimen 1) and surgery

*WHO, World Health Organization; BU, Buruli ulcer.

Patient 3 had lesions on both knees; he liked gardening and often knelt on soil and organic mulch that contained wood shavings. He first received rifampin and ethambutol for 5 weeks. Drug therapy was stopped because of hepatotoxicity. Trimethoprim-sulfamethoxazole and ciprofloxacin were then administered for 15 days. After completing oral therapy, he treated his lesions with a rifampin spray. Eight months after the start of drug therapy, the lesions were almost closed. A small ulcer remained on the right knee. The patient showed complete remission of lesions without any surgical intervention on his last control visits, 3 and 5 years after diagnosis.

Patient 4 had a medical history suggestive of leishmaniasis, and a smear from an ulcerated lesion was reported as positive for *Leishmania*. She was treated with intramuscular and intralesional sodium stibogluconate, including multiple inoculation sites. One month after receiving a course of 29 intramuscular injections, she showed more lesions in the left gluteal region and was treated with herbal medicine. BU was diagnosed from purulent material removed with a syringe and needle from a closed lesion. After drainage and biopsy her lesions showed improvement, and therefore no other treatment was instituted. She continued herbal treatment until total cure. The lesions have remained inactive for 5 years.

**Figure 2:**
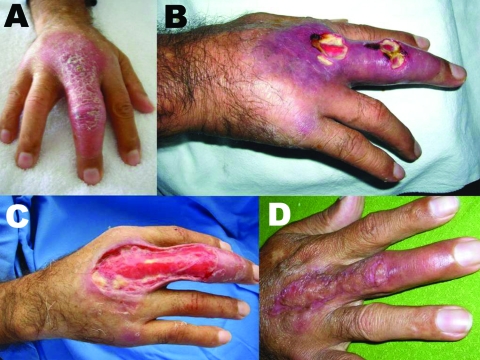
Patient 8. A) Nonulcerative edematous lesion on the right middle finger as first seen; B) ulcerated lesions on the right middle finger ≈4 weeks later; C) extensive debridement, 5.5 weeks after first seen; D) cured lesion 5 months after first seen, 1 month after autologous skin graft.

Patient 6 was treated by excision surgery and later received antituberculosis treatment (regimen 1) for 6 months. Patient 7 was pregnant when first seen. After tissue specimens were taken and BU was diagnosed, she was treated conservatively with topical disinfectants until delivery. She then received the WHO-recommended rifampin and streptomycin treatment (*12*) for 31 days and had excision surgery of the largest lesions. Later, all lesions were excised. Patient 8 had lesions on the right middle finger (Figure 2, **panel A**) that ulcerated after treatment for 1 month with ciprofloxacin, clindamycin, and dexametasone (Figure 2, **panel B**). Diagnosis was made on the basis of material obtained at the first extensive debridement (Figure 2, **panel C**). He received 5 weeks of regimen 1 treatment for tuberculosis, to which streptomycin was added for the last 3 weeks before surgical debridement and autologous skin graft. Antituberculous regimen 1 was continued for 2 more weeks. Figure 2, **panel D** shows the lesion 1 month after surgery. The patient then received 4 months of treatment with minocycline, ciprofloxacin, and trimethoprim-sulfamethoxazole and undertook rehabilitation including exercises. He recovered very good use of his right hand.

Patients 6, 7, and 8 were cured with antimicrobial agents and surgery. They had no recurrence after 2 years of follow-up.

As indicated in Table 2, all patients’ cases were confirmed by at least 2 positive laboratory tests. Seven patients showed acid-fast bacilli (AFB) on the initial smear, and 1 was negative (patient 5). The biopsy specimens from all the patients had AFB in histopathologic sections and typical histologic lesions, i.e., necrosis of fat and an abundance of extracellular clumps of AFB. Most biopsy specimens showed little or no inflammatory infiltrate. A granulomatous infiltrate was seen in the biopsy specimen from patient 5. Cultures remained negative, and IS*2404* PCR was positive for all 7 patients tested.

**Table 2:** Diagnostic laboratory studies on patients with Buruli ulcer, Peru*

Patient no.	ITM no.	AFB in smear (ZN)	Histopathologic changes	AFB in histologic sections	IS*2404* PCR	Culture
1	None	Positive	Necrosis of fat	Positive	ND	ND
2	96–0729	Positive	Necrosis of fat	Positive	Positive	Contaminated
3	01–0720	Positive	Necrosis of fat	Positive	Positive	Negative
4	02–1536	Positive	Necrosis of fat	Positive	Positive	Contaminated
5	02–1877	Negative (only 1 AFB seen)	Necrosis of fat + granulomatous infiltrate	Positive	Positive	Negative
6	04–0872	Positive	Necrosis of fat	Positive	Positive	Negative
7	05–2249 05–2411	Positive	Necrosis of fat + inflammation	Positive	Positive	Negative
8	None	Positive	Necrosis of fat + inflammation	Positive	Positive	Negative

*ITM, Institute of Tropical Medicine; AFB, acid-fast bacilli; ZN, Ziehl-Neelsen staining; ND, not done.

## Discussion

From 1969 until 2007, only 11 cases of BU have been reported in Peru, but no countrywide survey has been conducted to evaluate its true prevalence in Peru. BU is probably both infrequent and underreported in Peru and may often be misdiagnosed as leishmaniasis, which is more prevalent and better known. Three separate surveys suggest the rarity of BU. In the first, Saldaña-Patiño reviewed 1,620 ulcers biopsied from 1969 to 1981 and found no other BU patients apart from the 3 he reported (*8*). Second, a preliminary epidemiologic survey was conducted in the general area of the Huallaga Basin close to Tarapoto; several leishmaniasis and vascular lesions were found in 4 communities of ≈4,000 inhabitants, but no BU cases were seen (N. Donaires, MD thesis). Finally, physicians performing populationwide cysticercosis research in Tumbes (on the north coast) included a questionnaire and physical examination of all skin ulcers at the time, using as a guide a booklet in Spanish that was provided to familiarize them with BU (*13*). No skin ulcers were seen in the population surveyed.

The scarcity of BU cases in Peru and Mexico may be due to a lower virulence of the mycobacteria and a better immune response of patients when they become infected by *M. ulcerans*. It is clearly not related to infrequent contact with contaminated water. As in Africa, populations living in the Amazon River Basin have frequent contacts with water for domestic activities. Similarly, the low incidence of BU in Peru does not seem to be related to the absence of *M. ulcerans* in the environment, since the IS*2404* PCR positivity of the environmental specimens from Peru (collected in Tarapoto, in the Huallaga River Basin) and Benin have given comparable results (14% positivity in Peru vs. 10%–20% positivity in Benin) (F. Portaels et al., unpub. data).

The distribution of BU in Peru and elsewhere is strongly associated with wetlands, especially those with slow-flowing or stagnant water (e.g., ponds, backwaters, and swamps) (*4*,*5*). All of our patients had contacts with swampy areas in the Amazon River Basin (5 patients) or on the northern coast (3 patients). The 3 previously reported patients (*8*) had been in contact with water bodies related to tributaries of the Amazon River. In Peru, therefore, BU is present in the Peruvian jungle (*14*) and other swampy regions of the north coast.

In our study, the age of patients when they were first seen with BU ranged from 18 to 58 years. *M. ulcerans* is seen mainly in children and young adults in other BU-endemic regions but may affect any age group (*4*,*5*,*15*). All patients except patient 8 had lesions on the lower limb (Table 1). A similar pattern has been reported in other countries (*4*,*5*,*15*).

Patients sought medical assistance ≈1 month after the first lesion appeared. The longest interval to final diagnosis was 8 months, which led to a very large lesion in patient 4, who was originally being treated for leishmaniasis.

In Africa, the stigma associated with BU appears to be important, and its mysterious nature is often attributed to witchcraft and curses (*16*–*18*). Such concerns were not voiced by any of our patients, as they all had actively sought medical help.

Beside patients lost to follow-up, the clinical outcome of all patients from Peru was favorable. One patient (patient 4) was cured with herbal medicine only. Several authors report that while some topical treatments may heal BU lesions (*4*,*19*), other lesions may heal spontaneously (*20*). Patient 4 may have healed spontaneously or because of herbal medicine. Surgical treatment alone, which was until recently the mainstay of clinical management of BU in BU-endemic areas (*4*) is not practiced in Peru. Surgery is always associated with antimycobacterial drug therapy. Several centers in Africa have started to treat patients with streptomycin and rifampin according to WHO guidelines (*12*), and a recent study indicates that after 8 weeks of drug therapy ulcers may heal without surgery (*21*). The patients with reported infections in Peru up to 1988 had a favorable response to antituberculous therapy, although their lesions were large (*7*). In our study, patient 3 was also successfully treated with drug therapy without surgery. The success of antimycobacterial therapy in some areas may be correlated with a lower virulence of the *M. ulcerans* strains and in particular with lower production of mycolactones. African strains, which produce the greatest number and quantity of mycolactones, are associated with more severe disease forms (*22*), which may explain the difficulty treating some patients with only antimycobacterial drugs.

All patients in our study were PCR positive, but we were unable to cultivate *M. ulcerans* from the clinical specimens in Lima or Antwerp. This is surprising since the procedures used to cultivate *M. ulcerans* in primary culture were identical to those used for thousands of specimens from patients from other parts of the world (which yielded 45% of positive primary cultures) (*11*). Peruvian *M. ulcerans* strains may have different growth requirements or may be more sensitive to the antimicrobial agents in semisolid transport medium (PANTA: polymixin B, amphothericin B, nalidixic acid, trimethoprim, and azlocillin) than those from other geographic locations.

In conclusion, our study confirms that, although infrequently diagnosed, BU is an endemic disease in tropical swampy areas of Peru. Proper diagnosis and treatment require inclusion of simple clinical and laboratory guidelines in tuberculosis, leprosy, and leishmaniasis control programs, which reach health workers at all levels. Known BU-endemic areas should receive special emphasis. Education of populations and training of health workers are first needed to evaluate and understand the full extent of this disease in Peru.
